# Draft genomes of norovirus from stool samples of under-five children presenting with gastroenteritis in Malawi from 2012 to 2024

**DOI:** 10.1128/mra.00602-25

**Published:** 2025-07-28

**Authors:** Ernest Matambo, Flywell Kawonga, Chimwemwe Mhango, End Chinyama, Josephine Msowoya, Clara Majengo, Sesiyanda Maseko, Nkosazana Shange, Surprise Baloyi, Milton T. Mogotsi, Francis E. Dennis, Celeste Donato, Benjamin Kumwenda, Martin M. Nyaga, Chrispin Chaguza, Khuzwayo C. Jere

**Affiliations:** 1 Department of Pharmacy, School of Life Sciences and Allied Health Professions, Kamuzu University of Health Sciences 37610, Blantyre, Malawi; 2 Malawi-Liverpool-Wellcome Programme, Queen Elizabeth Central Hospital 299619, Blantyre, Malawi; 3 Institute of Infection, Veterinary and Ecological Sciences, University of Liverpool 4591, Liverpool, United Kingdom; 4 Biomedical Sciences Department, School of Life Sciences and Allied Health Professions, Kamuzu University of Health Sciences 37610, Blantyre, Malawi; 5 Department of Medical Laboratory Sciences, School of Life Sciences and Allied Health Professions, Kamuzu University of Health Sciences 37610, Blantyre, Malawi; 6 Next Generation Sequencing Unit, School of Biomedical Sciences and Division of Virology, Faculty of Health Sciences, University of Free State 37702, Bloemfontein, South Africa; 7 Department of Electron Microscopy and Histopathology, Noguchi Memorial Institute for Medical Research, University of Ghana 58835, Accra, Ghana; 8 Enteric Diseases Group, Murdoch Children’s Research Institute, Melbourne, Victoria, Australia; 9 Department of Paediatrics, The University of Melbourne 569523, Parkville, Victoria, Australia; 10 Department of Epidemiology of Microbial Diseases, Yale School of Public Health, Yale University 198926 , New Haven, Connecticut, USA; 11 NIHR Mucosal Pathogens Research Unit, Division of Infection and Immunity, University College London 308348 , London, United Kingdom; 12 NIHR Health Protection Research Unit in Gastrointestinal Infections, University of Liverpool 4591, Liverpool, United Kingdom; Queens College Department of Biology, Queens, New York, USA

**Keywords:** noroviruses, Malawi, whole genome, gastroenteritis

## Abstract

Norovirus is one of the most important etiological agents of gastroenteritis (GE) and food-borne diarrhea in all age groups worldwide. Here we report five draft genomes of norovirus isolated from stool samples collected from under-five children presenting with GE in Malawi from 2012 to 2024.

## ANNOUNCEMENT

Despite being a single-stranded RNA virus, norovirus is stable, highly infectious, and has been associated with gastroenteritis (GE) and food-borne diarrhea in all age groups globally (1–3). Norovirus (genus, *Norovirus* and family, *Caliciviridae*) has an approximately 7.5 kb genome with three open reading frames that encode a polyprotein (~5,100 bp), a viral protein 1 (~1,600 bp), and a viral protein 2 (~720 bp) (4–6). There are 10 known norovirus genogroups: GI–GX (7). Genogroups GI and GII primarily infect humans, whereas GIV, GVIII, and GIX rarely infect humans (3, 4, 7). In Malawi, norovirus is the third most prevalent viral etiological agent of GE but data on its genomic characterization are unavailable (8, 9). As part of the Sequencing and Antigenic Cartography of Enteric Viruses project, we randomly selected 10 archived stool samples per month from 2012 to 2024 that were collected from children of age less than 5 years who presented with GE at Queen Elizabeth Central Hospital for norovirus detection and sequencing. We report five draft genome sequences of the norovirus-positive RNA extracts that were successfully sequenced.

RNA was extracted from stool samples using the QIAamp Fast DNA Stool Mini Kit (Qiagen, Hilden, Germany) according to the manufacturer’s instructions. Norovirus was detected through real-time polymerase chain reaction using custom-designed enteric TaqMan Array Cards in methods as previously described (8, 10). The norovirus-positive stool samples were re-extracted using QIAamp RNA Mini kit (Qiagen, Hilden, Germany) and then quantified on a Qubit fluorometer using a High Sensitivity ssRNA Assay kit (Thermo Fisher Scientific, USA). cDNA was synthesized through whole transcriptome amplification using a Qiagen FX Whole Transcriptome Amplification kit. Genomic libraries were prepared using the Illumina DNA Prep kit (Illumina, USA) before being sequenced on Illumina NextSeq 2000 platform using a P1 flow cell and 300-cycle reagent kit (2 × 150  bp paired-end reads).

**Fig 1:**
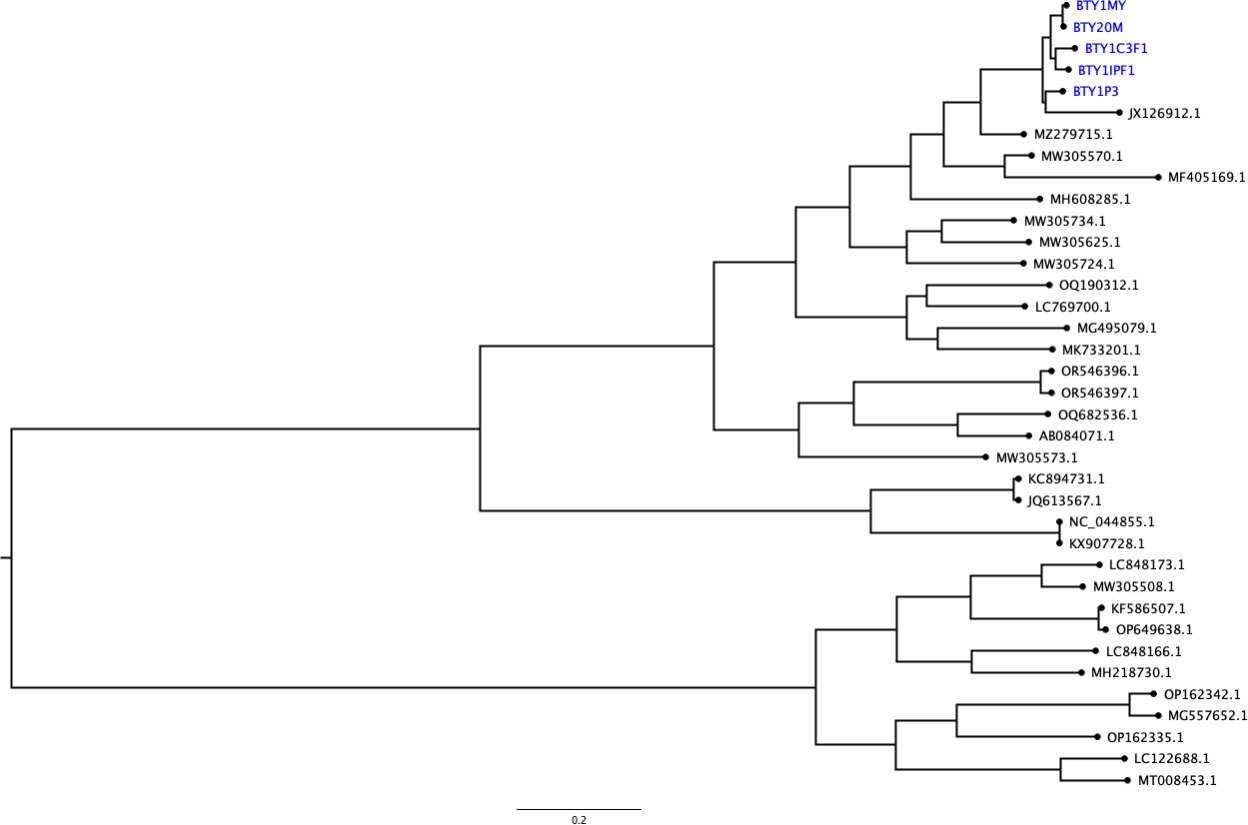
Maximum likelihood phylogenetic tree of 1% of 3,683 human norovirus global sequences and the five Malawi draft norovirus genomes (in blue, BTY1C3F1: PV611445, BTY1IPF1: PV611446, BTY1MY: PV611447, BTY1P3: PV611448, BTY20M: PV611449) subsampled based on phylogenetic diversity using Environment for Tree Exploration 3.1.3 (ETE3) toolkit (19). The global sequences were downloaded from NCBI using data sets 18.1.0 (https://www.ncbi.nlm.nih.gov/datasets/docs/v2/command-line-tools/). Multiple sequence alignment was performed using MAFFT ver.7, alignment curation was performed using trimAl v1.5.rev0 using automated parameters and visualized in Seaview 5.0.5 (20–22). The phylogenetic tree was constructed using IQ-TREE 2.3.6 with 1,000 bootstraps, visualized in FigTree v1.4.4 (http://tree.bio.ed.ac.uk/software/figtree/) and was midpoint rooted (23). The Malawi genomes clustered with published GII.17 and GII.4 strains (MZ279715.1 and JX126912.1, respectively) from the USA, supporting their GII genogroup classification.

Reads were quality assessed using FastQC 0.11.7 (https://www.bioinformatics.babraham.ac.uk/projects/fastqc/), trimmed using Trimmomatic 0.39, and their quality parameters collated using MultiQC 1.27.1 (11, 12). Contaminating human reads were removed by mapping to the human reference genome (GRCh38.p13) using Bowtie v2.5.4 (13). The non-human reads were mapped to the reference genome (JX459908.1) using the Burrows-Wheeler Alignment 0.7.18-r1243-dirty and variant calling was done using iVar 1.4.4 (14, 15). The assemblies were identified as norovirus using Genome Detective Virus Tool, genogroups assigned using a genotyping tool (https://www.rivm.nl/mpf/typingtool/norovirus/) and annotated using Prokka 1.14.6 (16, 17). Assembly completeness and identity were calculated using CheckV 1.0.3 and needle in EMBOSS 6.6.0.0 (http://emboss.open-bio.org/) respectively (18). Table 1 and Fig. 1 summarize the assembly characteristics and illustrate the phylogeny of the assemblies and the global genomes. The assemblies showed low divergence among the strains, possibly due to recent evolution from a common ancestor.

**TABLE 1:** Norovirus genome assembly parameters and their values

	BTY1P3	BTY1C3F1	BTY1IPF1	BTY1MY	BTY20M
Sample	Stool	Stool	Stool	Stool	Stool
Date of collection	26 September 2016	19 June 2015	05 October 2016	11 August 2016	23 August 2017
Ct values	26.379	28.448	23.855	28.454	34.332
Sequencing platform	Illumina NextSeq 2000	Illumina NextSeq 2000	Illumina NextSeq 2000	Illumina NextSeq 2000	Illumina NextSeq 2000
Reads	Paired	Paired	Paired	Paired	Paired
Average read length (trimmed)	119.21	120.41	128.47	124.53	124.17
Average sequencing depth	267.3×	754.3×	992.9×	183.3×	1115.8×
Average sequencing coverage (%)	99.9	97.8	99.9	98.3	99.7
Reference	JX459908.1	JX459908.1	JX459908.1	JX459908.1	JX459908.1
GC (%)	49.84	49.47	49.99	49.41	49.35
Assembly length	7,496	7,379	7,547	7,420	7,524
Completeness (%)	99.1	97.55	99.77	98.1	99.47
Sequence identity (%)	95.7	91.4	94.7	92.5	94.3
Genogroup	GII	GII	GII	GII	GII

## Data Availability

The genome sequences have been deposited in Genbank. Corresponding raw reads are available under the accession numbers SRX29148845 - SRX29148849 in the SRA database. The study was approved by the Malawi National Health Science Research Committee (NHSRC # 867).
